# Cigarette smoking reduced renal function deterioration in hypertensive patients may be mediated by elevated homocysteine

**DOI:** 10.18632/oncotarget.13308

**Published:** 2016-11-11

**Authors:** Feifei Huang, Jie Chen, Xun Liu, Feng Han, Qingqing Cai, Guicheng Peng, Kun Zhang, Weiqing Chen, Jingfeng Wang, Hui Huang

**Affiliations:** ^1^ Guangdong Provincial Key Laboratory of Malignant Tumor Epigenetics and Gene Regulation, Department of Cardiology, Sun Yat-Sen Memorial Hospital, Sun Yat-Sen University, Guangzhou, China; ^2^ Laboratory of RNA and Major Diseases of Brain and Heart, Sun Yat-Sen Memorial Hospital, Sun Yat-Sen University, Guangzhou, Guangdong, China; ^3^ Department of Radiation Oncology, Sun Yat-Sen Memorial Hospital of Sun Yat-Sen University, Guangzhou, China; ^4^ Division of Nephrology, Department of Internal Medicine, The Third Affiliated Hospital of Sun Yat-Sen University, Guangzhou, China; ^5^ Department of Ultrasound, State Key Laboratory of Oncology in Southern China, Sun Yat-Sen University Cancer Center, Guangzhou, China; ^6^ Department of Medical Oncology, Sun Yat-Sen University Cancer Center, Sun Yat-Sen University, Guangzhou, China; ^7^ Department of Internal Medicine, Dongguan Hengli Hospital, Dongguan, China; ^8^ Department of Biostatistics and Epidemiology, School of Public Health, Sun Yat-Sen University, Guangzhou, China

**Keywords:** cigarette smoking, hypertension, renal function deterioration, homocysteine

## Abstract

Elevated homocysteine (HCY) and smoking are both important risk factors for hypertensive patients. However, whether they have crossing effect on renal function deterioration of hypertensive patients and what is the underlying mechanism are unclear. In the present study, 3033 participants diagnosed as essential hypertension with estimated glomerular filtration rate (eGFR)> 30 ml/min/1.73 m^2^ from southern China were enrolled in this cross-sectional study. We collected the demographic and clinical data. In addition, the mediation effects were analyzed. The results showed that, comparing with non-smokers, smokers had significant higher levels of HCY (13.10 (11.20−16.87) *vs*. 11.00 (8.90−13.40) umol/L, *P* < 0.001) and lower eGFR (79.71 (66.83−91.05) *vs*. 82.89 (69.80−95.85) ml/min/1.73m^2^, *P* < 0.001). HCY levels and smoking were independently associated with decreased eGFR. Meanwhile, eGFR levels were significantly negatively correlated with HCY (*P* < 0.001), and this correlation might be stronger in current smokers. Current smoker consuming over 20 cigarettes per day would accelerate early renal function deterioration (OR = 1.859, *P* = 0.019). The mediation effects analysis further showed that the association between smoking and renal function deterioration was mediated by HCY. And elevated HCY was accounted for 56.94% of the estimated causal effect of smoking on renal function deterioration in hypertensive patients. Our findings indicated that cigarette smoking was associated with renal function deterioration in hypertensive patients, and the association between cigarette smoking and renal function deterioration was probably mediated by elevated HCY. Therefore, HCY-lowering therapy may be beneficial for renal function deterioration in hypertensive smoking patients.

## INTRODUCTION

Chronic kidney disease (CKD) is a worldwide public health problem, and it independently increases the risk of cardiovascular diseases (CVD) [1, 2]. Estimated glomerular filtration rate (eGFR) is usually used for assessing the severity of CKD [3]. Previous studies have found that reduced eGFR is associated with a high prevalence of CVD among the general population [4, 5], especially among high-risk patient populations with existing hypertension [2, 6]. However, it is still lack of effective therapy for renal function deterioration in hypertensive patients. Thus, it remains urgent to explore the key risk factors of reduced eGFR in hypertensive patients.

Cigarette smoking has being prevalent in economically developing regions of the world, especially in China [7, 8]. China is the world’s largest consumer of tobacco, and 52.9% of men are smokers [9, 10]. Due to these factors, China is burdened by smoking-related diseases that result in nearly one million deaths each year [11, 12]. It has been reported that cigarette smoking significantly increased the risk of reduced eGFR in patients with type 2 diabetes [13]. And former smokers generally had a significantly lower eGFR than never smokers from 6 months after lung transplantation [14]. All these studies indicated that cigarette smoking is an independent risk factor for CKD. However, the underlying mechanisms for these associations have not been clarified.

Homocysteine (HCY) is a toxic non-proteinogenic amino acid biosynthesized from methionine [15]. Previous studies demonstrated that elevated HCY was highly prevalent and significantly related to cardiovascular morbidity and mortality in patients with CKD [16, 17]. Both active smoking and passive smoking were demonstrated to be positively correlated with increased serum HCY levels [18, 19]. Moreover, it has been demonstrated that HCY not just worked as a marker for renal damage, it could induce chronic inflammation and promote glomerulosclerosis [20]. However, there is still lack of direct evidence to show that cigarette smoking reduces eGFR by increasing serum HCY levels. In this study, we hypothesized that HCY mediated the association between cigarette smoking and reduced eGFR.

## RESULTS

### Baseline characteristics of smokers and nonsmokers

789 participants were excluded for primary renal disease, history of hypertension, secondary hypertension, antihypertensive drugs usage, and related vitamins usage. 3033 participants diagnosed as essential hypertension with eGFR > 30 ml/min/1.73m^2^ were finally enrolled in the study. Demographic and clinical characteristics of the study population were presented in Table 1. Overall, 799 participants were smokers (601current smokers and 198 former smokers), and 2234 participants were non-smokers.

**Table 1:** Clinical characteristics of subjects in Smoking and No smoking groups

	Smoking (*N* = 799)	No smoking (*N* = 2234)	*P* value
**Population characteristics**			
Age (years)	61 (55–68)	62 (55–70)	0.177
Sex (M/F)	771/28	447/1787	< 0.001*
**Physical measurements**			
SBP (mmHg)	146 (134–159)	147 (136–161)	0.122
DBP (mmHg)	85 (76–92)	82 (74–88)	< 0.001*
**Serum biochemical results**			
HCY (umol/L)	13.10 (11.20–16.87)	11.00 (8.90–13.40)	< 0.001*
BUN (mmol/L)	5.75 (5.00–6.68)	5.70 (4.97–6.60)	0.014*
Scr (umol/L)	88.80 (78.85–103.58)	70.30 (60.60–82.70)	< 0.001*
eGFR (mL/min per 1.73 m^2^)	79.71 (66.83–91.05)	82.89 (69.80–95.85)	< 0.001*
UA (umol/L)	423 (343–494)	366 (310–430)	< 0.001*
**Urinary biochemical results**			
Urinary albumin (mg/L)	10.00 (5.00–27.90)	9.30 (5.00–27.13)	0.340
Urinary creatinine (mmol/L)	9.10 (6.60–12.78)	7.10 (4.80–10.60)	< 0.001*
ACR (mg/mmol)	1.50 (0.90–3.90)	1.10 (0.70–2.60)	< 0.001*

Abbreviations: Value presented as median [interquartile range (IQR)].

* Significant difference between Smoking group and No smoking group (*P* < 0.05).

ACR, urinary albumin to creatinine; BUN, blood urea nitrogen; DBP, diastolic blood pressure; eGFR, estimated glomerular filtration rate; HCY, homocysteine; SBP, systolic blood pressure; Scr, serum creatinine; UA, uric acid.

On average, smokers were more likely to be man, while age was similar between smokers and non-smokers. Smokers had higher diastolic blood pressure (DBP) than non-smokers (*P* < 0.05). However, there was no difference in the levels of systolic blood pressure (SBP) between smokers and non-smokers (*P* > 0.05).

As for the renal function, smokers had significantly higher blood urea nitrogen (BUN), uric acid (UA), serum creatinine (Scr), urinary creatinine and the ratio of urinary albumin to creatinine (ACR) comparing with non-smokers (*P* < 0.05). Interestingly, comparing with non-smokers, smokers had lower eGFR (79.71(66.83–91.05) *vs*. 82.89 (69.80–95.85) ml/min/1.73m^2^, *P* < 0.001). However, there was no significant difference of urinary albumin between smokers and non-smokers(*P* > 0.05).

In addition, comparing with non-smokers, smokers had significant higher levels of HCY (13.10 (11.20–16.87) *vs*. 11.00 (8.90–13.40) umol/L, *P* < 0.001).

### Independent influencing factors for renal function deterioration

To further explore the independent influencing factors of renal function deterioration in hypertensive patients, parameters which may be relate to reduced eGFR (in the Table 1) were enrolled into binary logistic regression analysis. The analysis showed that sex (B = 0.724, *P* < 0.001), smoking (B = −0.463, *P* = 0.013), HCY (B = −0.084, *P* = 0.018) and Scr (B = 1.244, *P* < 0.001) were all independently associated with eGFR (Table 2).

**Table 2:** The binary logistic regression analysis of independent risk factors of low eGFR levels by an adjusted model

	B	OR	95% CI	*P* value
Age	−0.002	0.998	0.990–1.005	0.569
Sex	0.724	2.063	1.857–2.292	< 0.001*
(Male = 1, Female = 0)				
Smoking	−0.463	0.629	0.437–0.906	0.013*
(Smoking = 1, No smoking = 0)				
SBP	0.008	1.008	0.990–1.028	0.379
DBP	−0.008	0.992	0.968–1.017	0.544
HCY	−0.084	0.919	0.857–0.986	0.018*
BUN	−0.023	0.977	0.623–1.532	0.919
Scr	1.244	3.468	2.895–4.155	< 0.001*
UA	−0.001	0.999	0.994–1.004	0.707
Urinary albumin	−0.001	0.999	0.988–1.010	0.905
Urinary creatinine	−0.017	0.983	0.899–1.076	0.712
ACR	−0.066	0.937	0.782–1.122	0.477

Abbreviations: ACR, urinary albumin to creatinine; BUN, blood urea nitrogen; DBP, diastolic blood pressure; eGFR, estimated glomerular filtration rate; HCY, homocysteine; SBP, systolic blood pressure; Scr, serum creatinine; UA, uric acid.

* *P* < 0.05.

### Relationship among HCY, renal function deterioration and smoking status

**Figure 1:**
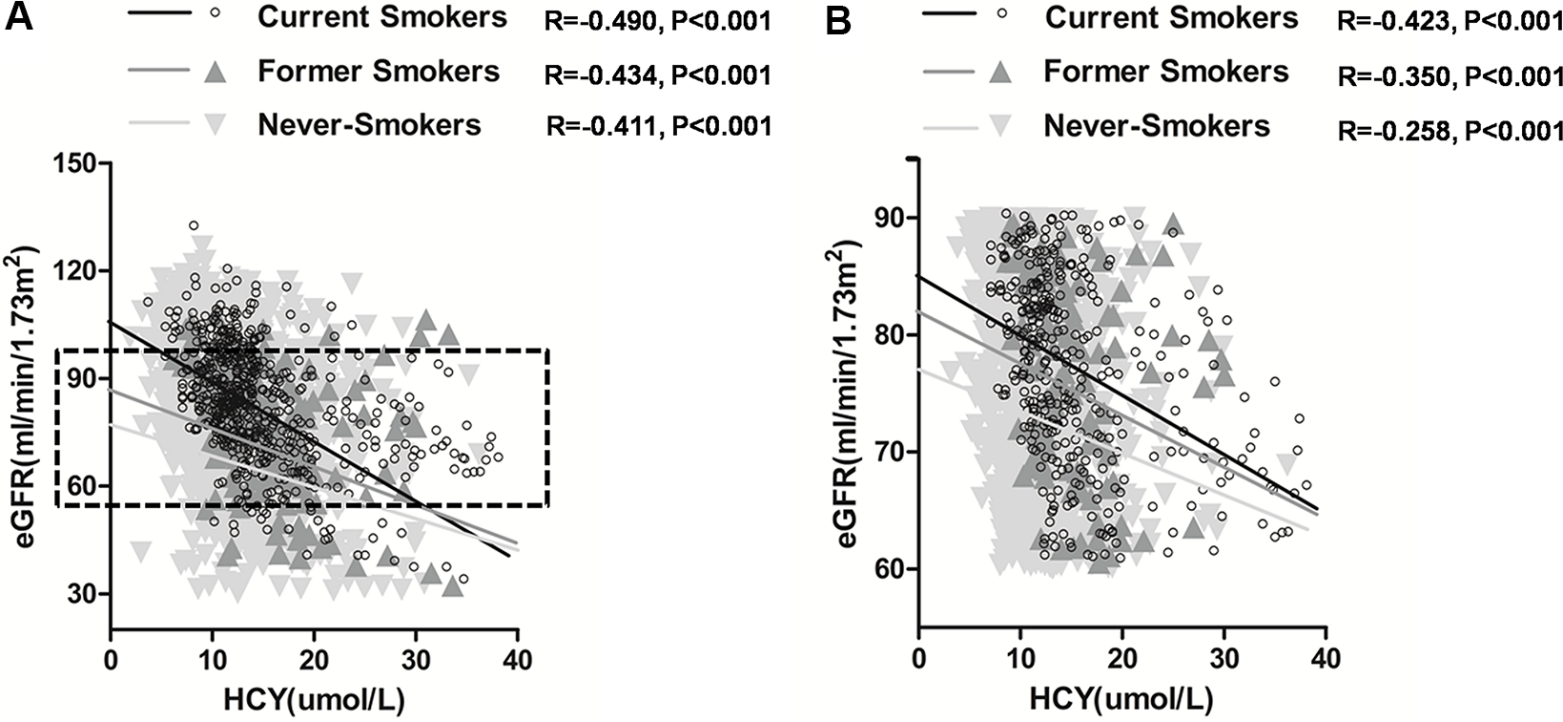
(**A**) Correlation between homocysteine (HCY) and estimated glomerular filtration rate (eGFR) in current smokers group, former smokers group, and never-smokers group respectively. (**B**) Correlation between HCY and eGFR in subjects with 60 ml/min/1.73 m^2^ ≤ eGFR < 90 ml/min/1.73 m^2^ classified by current smoking, former smoking, and never-smoking respectively.

We also explored the relationship among HCY, eGFR and smoking status in all enrolled subjects after the adjustment for the potential confounding factors (Table 3). There was a significant correlation between HCY and eGFR in current smokers (*r* = −0.490, *P* < 0.001), and this correlation might be stronger than in former smokers (*r* = −0.434, *P* < 0.001) and never-smokers (*r* = −0.411, *P* < 0.001) (Figure 1A) presumably.

**Table 3:** Correlations between HCY and eGFR in current smokers group, former smokers group, and never-smokers group respectively

eGFR (mL/min per 1.73 m^2^)	group	*r*	*P* value
30 ≤ eGFR (*n* = 3033)	Current smokers (*n* = 601)	−0.490	< 0.001*
	Former smokers (*n* = 198)	−0.434	< 0.001*
	Never-smokers (*n* = 2234)	−0.411	< 0.001*
60 ≤ eGFR < 90 (*n* = 1590)	Current smokers (*n*= 363)	−0.423	< 0.001*
	Former smokers (*n*= 113)	−0.350	< 0.001*
	Never-smokers (*n*= 1114)	−0.258	< 0.001*

Abbreviations: eGFR, estimated glomerular filtration rate; HCY, homocysteine.

* *P* < 0.05 was considered statistically significant.

Interestingly, we got the similar results of the correlation between HCY and eGFR in subjects with eGFR between 60 ml/min/1.73m^2^ and 90 ml/min/1.73m^2^. The correlation between HCY and eGFR in current smokers (*r* = −0.423, *P* < 0.001) might be stronger than in former smokers (*r* = −0.350, *P* < 0.001) and never-smokers (*r* = −0.258, *P* < 0.001) (Figure 1B) presumably.

### Relationship among HCY, renal function deterioration and daily cigarette consumption

**Figure 2:**
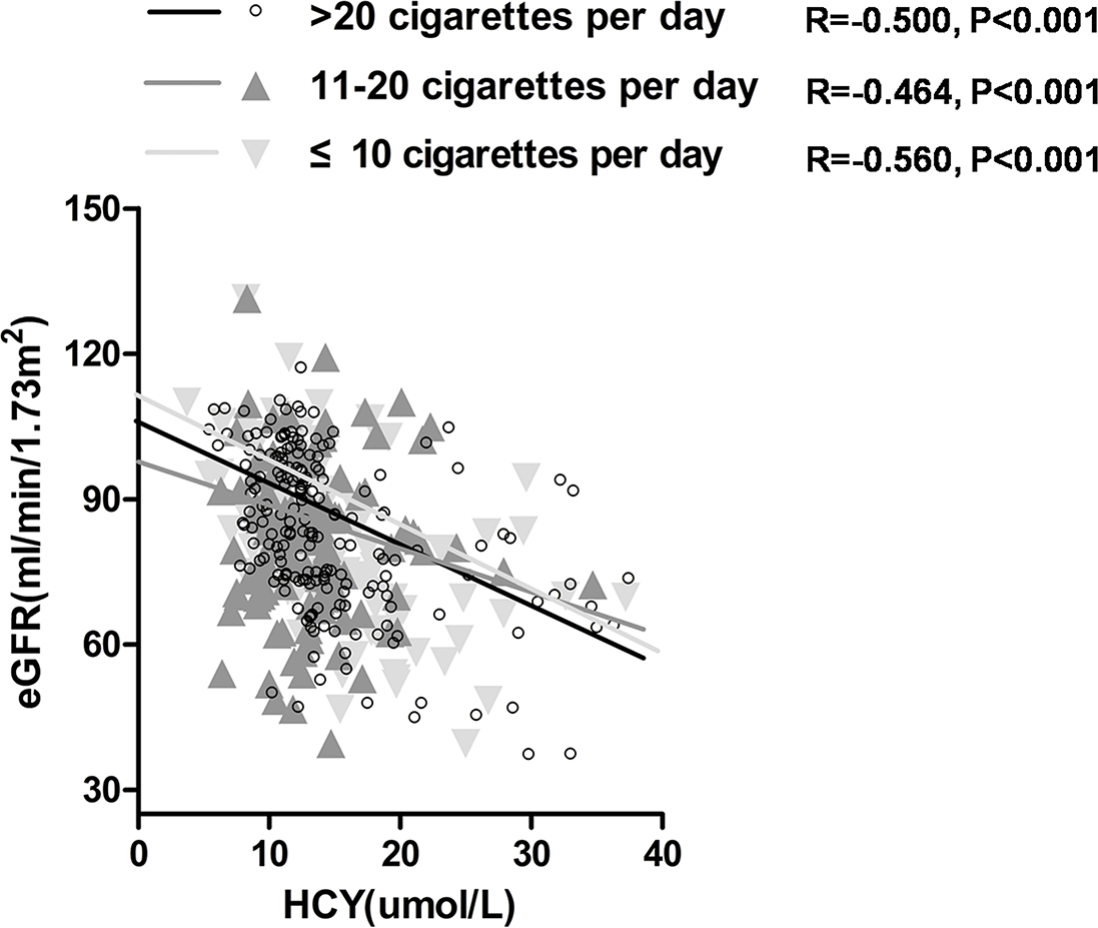
Correlation between homocysteine (HCY) and estimated glomerular filtration rate (eGFR) in current smokers consuming over 20 cigarettes per day group, 11–20 cigarettes per day group, and no more than 10 cigarettes per day group

Meanwhile, we also analyzed the relationship among HCY, eGFR and daily cigarette consumption in enrolled subjects after adjustment of age, sex, blood pressure (Table 4). eGFR levels was negatively correlated with HCY in different cigarette consumption (smokers consuming over 20 cigarettes per day (*r* = −0.500, *P* < 0.001), 11–20 cigarettes per day (*r* = −0.464, *P* < 0.001) and no more than 10 cigarettes (*r* = −0.560, *P* < 0.001)) (Figure 2).

**Table 4:** Correlations between HCY and eGFR in current somkers consuming over 20 cigarettes per day group, 11–20 cigarettes per day group, and no more than 10 cigarettes per day group

Amount of cigarette smoked per day	*r*	*P* value
≤ 10 (*n* = 118)	−0.560	< 0.001*
11–20 (*n* = 289)	−0.464	< 0.001*
> 20 (*n* = 194)	−0.500	< 0.001*

Abbreviations: eGFR, estimated glomerular filtration rate; HCY, homocysteine.

* *P* < 0.05 was considered statistically significant.

**Figure 3:**
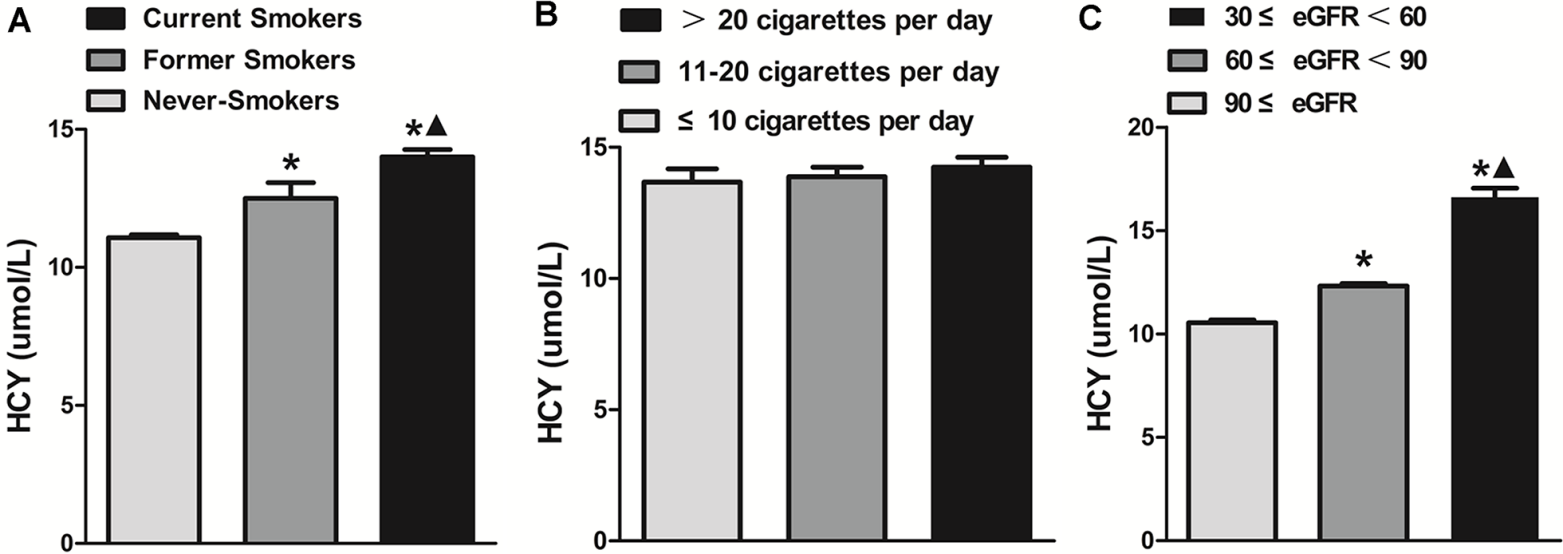
(**A**) The serum homocysteine (HCY) levels in current smokers group, former smokers group, and never-smokers group. *P < 0.05 *vs*. Never-Smokers; ▲*P* < 0.05 *vs*. Former Smokers. (**B**) The serum homocysteine (HCY) levels in current smokers consuming over 20 cigarettes per day group, 11–20 cigarettes per day group, and no more than 10 cigarettes per day group. (**C**) The serum homocysteine (HCY) levels in subjects with estimated glomerular filtration rate (eGFR) ≥ 90 ml/min/1.73m^2^ group, 60 ml/min/1.73 m^2^ ≤ eGFR < 90 ml/min/1.73 m^2^ group, and 30 ml/min/1.73 m^2^ ≤ eGFR < 60 ml/min/1.73 m^2^ group. *P < 0.05 *vs*. eGFR ≥ 9 0 ml/min/1.73 m^2^ group; ▲*P* < 0.05 *vs*. 60 ml/min/1.73 m^2^ ≤ eGFR < 90 ml/min/1.73 m^2^ group.

**Figure 4:**
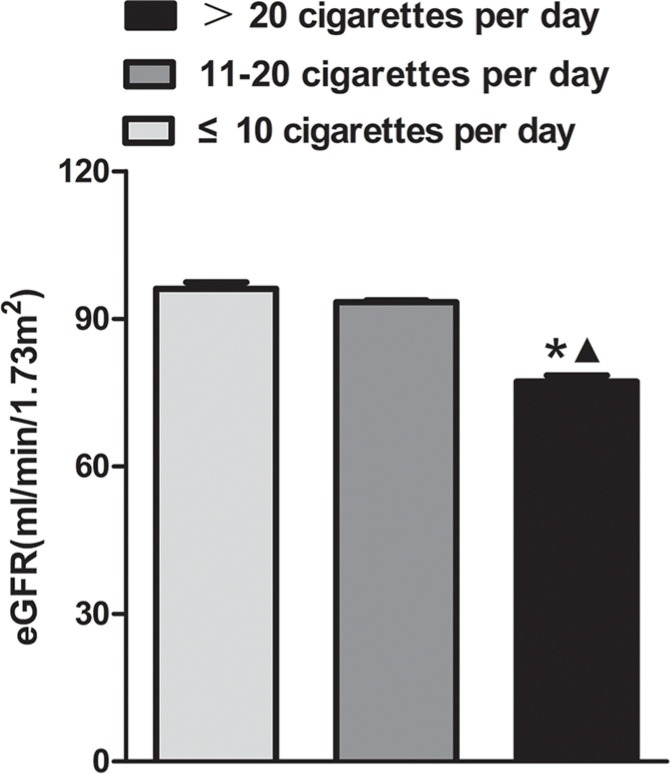
The estimated glomerular filtration rate (eGFR) levels in current smokers consuming over 20 cigarettes per day group, 11–20 cigarettes per day group, and no more than 10 cigarettes per day group *P < 0.05 *vs*. no more than 10 cigarettes group; ▲*P* < 0.05 *vs*. 11–20 cigarettes per day group.

We detected serum HCY levels in subjects with three smoking status, different cigarette consumption per day and different renal function levels (Figure 3). We found that former smokers had significant higher levels of HCY than never-smokers (12.50 (9.50–15.80) *vs*. 11.00(8.90–13.40) umol/L, *P* < 0.001). Current smokers had significant higher levels of HCY (14.00 (10.80–15.30) *vs*. 12.50 (9.50–15.80) umol/L, *P* = 0.02), comparing with former smokers (Figure 3A). However, there was no significant difference of HCY levels among the current smokers consuming over 20 cigarettes per day (14.24 (10.90–15.90) umol/L), 11–20 cigarettes per day (13.88 (10.50–15.30) umol/L), and no more than 10 cigarettes (13.67 (10.30–16.25) umol/L) (*P* > 0.05) (Figure 3B). Subjects with 60 ml/min/1.73m^2^ ≤ eGFR < 90 ml/min/1.73m^2^ had significant higher levels of HCY than those with eGFR ≥ 90 ml/min/1.73m^2^ (12.32(9.50–13.90) *vs*. 10.54 (8.00–12.00) umol/L, *P* < 0.001). And Subjects with 30 ml/min/1.73m^2^ ≤ eGFR <60 ml/min/1.73m^2^ had significant higher levels of HCY than those with 60 ml/min/1.73m^2^ ≤ eGFR < 90 ml/min/1.73m^2^ (16.61 (12.43–19.00) *vs*. 12.32 (9.50–13.90) umol/L, *P* < 0.001) (Figure 3C). Current smokers consuming over 20 cigarettes per day had lower eGFR levels (77.25 (66.62–93.37) ml/min/1.73m^2^) than those consuming 11–20 cigarettes per day (93.35 (89.24–95.44) ml/min/1.73m^2^) or no more than 10 cigarettes (96.10 (79.84–112.30) ml/min/1.73m^2^) (*P* < 0.001). However, there was no significant difference of eGFR levels between smoker consuming 11–20 cigarettes per day and those consuming no more than 10 cigarettes (*P >* 0.05) (Figure 4). In addition, in current smokers with 60 ml/min/1.73m^2^ ≤ eGFR < 90 ml/min/1.73m^2^ consuming over 20 cigarettes per day accelerated renal function deterioration (OR = 1.859, *P* = 0.019), and those who smoked 11–20 cigarettes per day had no such a significant effect on renal function deterioration (*P* = 0.290), compared with those who smoked 10 cigarettes or less per day (Table 5). However, the effect of daily cigarette consumption on renal function deterioration was not significant in smokers with 30 ml/min/1.73m^2^ ≤ eGFR< 60 ml/min/1.73m^2^.

**Table 5:** The associations between amount of cigarette smoked per day and eGFR in current smokers

Amount of cigarette smoked per day	90 ≤ eGFR	60 ≤ eGFR < 90			30 ≤ eGFR < 60		
		OR	95% CI	*P*	OR	95% CI	*P*
≤ 10	1			0.047*			0.312
11–20	1	1.308	0.759–2.153	0.290	1.086	0.469–2.512	0.848
> 20	1	1.859	1.106–3.124	0.019*	1.798	0.718–4.505	0.211

The adjusted OR was calculated by binary logistic regression models with adjustment for age, sex. eGFR, estimated glomerular filtration rate.

* *P* < 0.05.

### The mediation effects of elevated HCY between smoking and renal function deterioration

**Figure 5:**
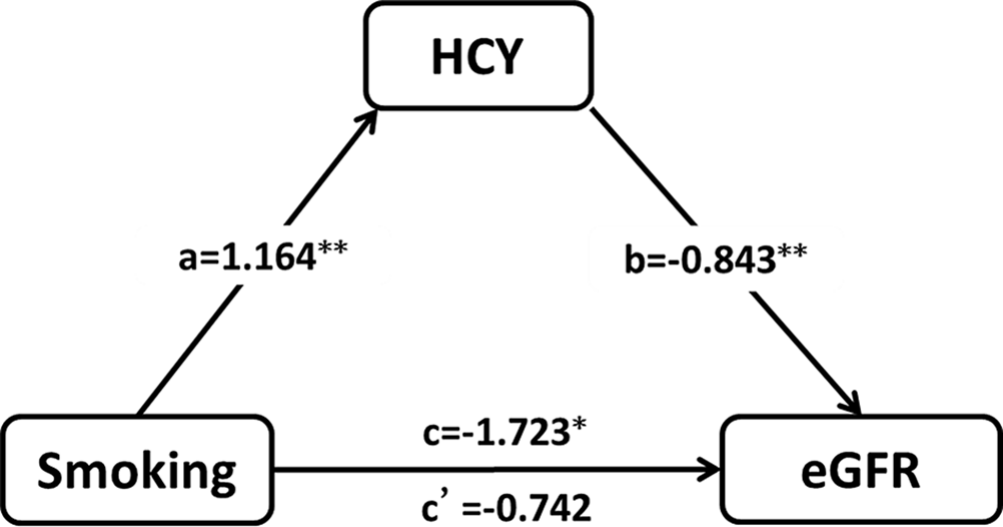
The mediation of homocysteine (HCY) on the association between smoking and deterioration of renal function in hypertensive patients (**P < 0.001, *P < 0.05)

In order to explore the exact relationship among HCY, smoking and renal function deterioration, we further performed the mediation effects analysis after adjustment of age, sex, blood pressure, glucose, lipids. Figure 5 provided an illustration of the simple mediation model between smoking and eGFR through HCY, and the results showed that HCY probably mediated the association between smoking and eGFR. The indirect effect of smoking on eGFR through HCY was significant (ab = −0.981, 95% bias-corrected (BC) bootstrap confidence interval (CI): (−1.480∼−0.529). The total effect of smoking on eGFR became smaller when potential mediator HCY was included in the model (total effect *c* = −1.723, *P* = 0.028; direct effect *c*′= −0.742, *P* = 0.329). These results demonstrated that the association between smoking and renal function deterioration in hypertensive patients was fully mediated by HCY, and the mediated effect size was (−0.981) / (−0.981–0.742) = 56.94%.

## DISCUSSION

A healthy lifestyle was associated with lower risk of coronary heart disease mortality [21], especially in individuals with CKD [22]. Smoking was one of the most significant risk factor associated with a high weighed health lifestyle [22, 23]. In the present study, we discovered that hypertensive patients with smoking suffered from significantly lower eGFR than non-smokers. Moreover, our study further demonstrated both cigarette smoking and HCY were independent risk factors for reduced eGFR in Chinese hypertensive patients. And the association between smoking and reduced eGFR was probably mediated by elevated HCY.

Several prior studies have reported that cigarette smoking, in a dose-dependent manner, was a risk factor for renal function deterioration [24, 25]. However, little information is available about the underlying mechanisms for these associations. Li [26] and Lu et al. [27] successively reported that elevated HCY levels was an important pathogenic factor for glomerular damage. Similar to previous studies, we also found that smoking and HCY worked as independent influencing factors for renal function deterioration in hypertensive patients. Moreover, our study further observed that eGFR were significantly negatively correlated with HCY, and this correlation might be stronger in current smokers. Besides, eGFR was also significantly correlated with HCY in different cigarette consumption respectively, and only smokers consuming over 20 cigarettes per day showed accelerating early renal function deterioration. Thus, HCY may involve in smoking related renal function deterioration. Meanwhile, the effect of HCY and smoking on renal function deterioration was notable in hypertensive patients with early renal function deterioration.

In the whole hypertensive patients of our study, cigarette consumption exerted a dose-dependent manner impact on renal function. And previous study also reported that cigarette smoking, in a dose-dependent manner, was identified as a key prognostic factor in IgA nephropathy [24]. In our study, we found that current smoker consuming over 20 cigarettes per day would accelerate early renal function deterioration (eGFR between 60–90 ml/min/1.73m^2^), the effect of daily cigarette consumption on renal function deterioration was not significant in smokers with 30 ml/min/1.73m^2^ ≤ eGFR < 60 ml/min/1.73m^2^. That may attribute to the other complicated physiological and pathological factors in the stage of 30 ml/min/1.73m^2^ ≤ eGFR < 60 ml/min/1.73m^2^ or end-stage renal disease. Therefore, smoking cessation may be more beneficial for early deterioration of renal function in hypertensive.

However, the underlying mechanism of renal function deterioration induced by cigarette smoking are not clear. We conducted the mediation analysis provided further insight into the mechanism of how HCY may impact the association between cigarette smoking and renal function deterioration.

Our study observed that HCY mediated the association between smoking and renal function deterioration in hypertensive patients and accounted for 56.94% of this association. This finding more fully confirmed our original hypothesis. The following reasons might explain the mediation between smoking and renal function deterioration. First, cigarette smoking is a significant source of free oxygen radical, which may alter the redox of mercapto (−SH) and cause the increase of plasma HCY levels [18]. In addition, cigarette smoke components could induce increased interleukin 6 (IL-6) production, which activates pyridoxal phosphatase in hepatocytes. Thus decreasing pyridoxal phosphatase level would result in a series of reactions, block the second homocysteine metabolic pathway and trans-sulphuration and be responsible, which could be another reason of increased HCY levels in smokers [18, 28–30]. Second, elevated level of HCY could induce chronic inflammation in vascular bed, including glomerulus, and promote glomerulosclerosis [20]. Furthermore, HCY may promote the differentiation of inflammatory monocyte subsets directly [31, 32], inducing a vicious circle of inflammatory process in the development of renal function deterioration. HCY may also function as an atherogenic factor by increasing oxidative stress, impairing endothelial function, and inducing thrombosis, all of these contribute to the development of renal function deterioration [33–36].

These findings in this study have important clinical implications. In the past 10 years, renal function deterioration has received increasing attention as a leading public health problem, especially its directly affects on cardiovascular events. Our study suggested that cigarette smoking and HCY are two nodes in a web of risk factors for renal function deterioration in hypertensive patients and that mediation effects potentially offer new opportunities for clinically intervening to reduce risk associated with one variable, making it easier to intervene on the other nodes in the pathogenic network of renal function deterioration in hypertensive patients.

This study firstly showed that HCY mediated the association between cigarette smoking and reduced eGFR in hypertensive patients. However, some potential limitations of our study should be noted. First, it is a cross-sectional study and the sample size was limited. Further researches are needed to detect the direct causal relationship between smoking and reduced eGFR in hypertensive patients. Second, smoking status was not validated using biochemical tests, such as measurements of cotinine and carbon monoxide. Self-reported smoking status might be biased. Third, the data for inflammatory and oxidative stress markers were not collected, therefore further studies should be carried out to make certain about the specific mechanism of inflammatory and oxidative stress on reduced eGFR.

In conclusion, cigarette smoking was associated with renal function deterioration in Chinese hypertensive patients, and the association between cigarette smoking and renal function deterioration was probably mediated by elevated HCY. This study emphasizes the importance of smoking cessation especially in patients with reduced eGFR. At the same time, the mediation effects potentially offer new opportunities for clinically intervening on smoking and HCY in the pathogenic network of reduced eGFR in hypertensive patients. So, in order to prevent deterioration of renal function, more attention should be paid to the smoking hypertensive patients, especially those with elevated HCY.

## MATERIALS AND METHODS

### Ethics statement

The study protocol conformed to the ethical guidelines of the 1975 Declaration of Helsinki as reflected in a priori approval by the Ethics Committee of Sun Yat-sen Memorial Hospital of Sun Yat-sen University. Informed written consent was obtained from each participant and their medical records were studied by anonymous means. And the consent procedure was approved by the Ethics Committee of Sun Yat-sen Memorial Hospital of Sun Yat-sen University.

### Study population and data collection

3822 essential hypertensive subjects (indigenous adult aged more than or equal to 35 years) from general population of 17 villages in southern China were enrolled in this cross-sectional study. Subjects with severe renal damage (eGFR ≤ 30ml/min/1.73m^2^), primary renal disease, and other diseases inducing renal damage including diabetes, multiple myeloma, vasculitis, systemic lupus erythematosus were excluded. Furthermore, subjects with history of hypertension, antihypertensive drugs usage, anemia history, and malnutrition (additional vitamins B6, B12 and folic acid usage) were also excluded.

### Questionnaire survey

Trained medical students or clinicians administered a standardized questionnaire to each subject. The demographic data (e.g. age, sex, etc.), smoking behaviors, personal and family health history (e.g. hypertension, diabetes, and renal disease) were collected during the survey. History of hypertension, antihypertensive drugs usage, smoking, CKD and nephrotoxic medications (e.g. non-steroidal anti-inflammatory drugs) were noted.

### Measurement and definition of smoking behaviors

All individuals were categorized into three groups: never smokers, current smokers and former smokers as previous report [37, 38]. Never smokers were defined as those who had never smoked at least 1 cigarette a day in one’s lifetime. Current smokers were defined as having smoked greater than 100 cigarettes in one’s lifetime and having smoked at least one cigarette daily for 30 days by the time of the interview. Former smokers were defined as those who had smoked at least 100 cigarettes over their lifetime, but had stopped by the past 30 days. Based on this information, a two-level smoking status variable was created: (i) smoking (current smokers and former smokers) and (ii) no smoking (never smokers). Every current smoker reported their average number of cigarettes consumed per day in their latest smoking duration. By cigarettes consumption the current smokers were categorized into three groups: light smokers (consuming 1–10 cigarettes per day), moderate smokers (consuming 11–20 cigarettes per day), and heavy smokers (consuming > 20 cigarettes per day) [39].

### Blood pressure measurement and diagnosis criteria for hypertension

Blood pressure was measured by a mercury sphygmomanometer and proper bladder, three times at 1 min intervals, with the participants in the sitting position after 5 minutes of rest. SBP and DBP were identified by phase I and V (disappearance) Korotkoff sounds. The mean of the three readings was calculated, unless the difference between the readings was greater than 10 mmHg, in which case the mean of the two closest measurements was used. Each smoker was asked not to smoke after getting up until finishing the BP measurement. Essential hypertension was defined as a SBP ≥ 140 mmHg, and or DBP ≥ 90 mmHg on three or more occasions in different time according to the 2007 European Society of Hypertension and European Society of Cardiology (ESH-ESC) Practice Guidelines. Additionally, secondary hypertension patients were excluded from this study [40].

### Biochemical data collection

We measured the levels of urinary creatinine, albumin. ACR was calculated (mg/mmol). In addition, we measured Scr and eGFR using the Chronic Kidney Diease Epidemiology Collaboration (CKD-EPI) equation (eGFR =141 × min(Scr/κ, 1)^α^ × max(Scr/κ, 1)^−1.209^ × 0.993^Age^ × 1.018[if female]_1.159[if black], where κ is 0.7 for females and 0.9 for males, α is –0.329 for females and –0.411 for males, min indicates the minimum of Scr/κ or 1, and max indicates the maximum of Scr/κ or 1 [41].

Venous blood samples were drawn from each subject after at least 10 hours of overnight fasting. BUN, and UA were performed with commercially available reagents. Serum HCY concentrations were determined using a commercially available fluorescence polarization immunoassay (FPIA) kit (IMx^®^ System Homocysteine, Abbott Laboratories, North Chicago, IL). All laboratory measurements met a standardization and certification program.

### Statistical analyses

Continuous data were expressed as mean ± standard deviation (SD) or median [interquartile range (IQR)]. Baseline characteristics were compared between smokers and non-smokers using the *t* test or Mann-Whitney *U* test. Categorical data were presented as percentages and were compared using the chi-square test or Fisher’s exact test. We analyzed the association between eGFR and relevant covariates with logistic regression models to evaluate independent parameters affecting the eGFR levels. Adjusted correlation between HCY and eGFR was evaluated by partial correlation analysis.

Mediation effects analysis: Mediation is demonstrated when the following conditions are met: the main in dependent variable (i.e. smoking) is significantly associated with the main dependent variable (i.e. eGFR); the independent variable (i.e. smoking) is significantly related with the mediator variable (i.e. HCY); and mediator variable (i.e. HCY) is significantly associated with the independent variable (i.e. eGFR) when the independent variable (i.e. smoking) is controlled for. The mediated effect size was evaluated by a formula ab/(ab+c′), [42] where *a* is the coefficient relating the independent variable the mediator, *b* is the coefficient relating the mediator to the dependent variable adjusted for the independent variable, *c*′ is the coefficient relating the independent variable to the dependent variable adjusted for the mediator. Potential confounding factors adjusted for in the multivariate analysis included age, sex, blood pressure, glucose and lipids.

All statistical analyses were performed using the software SPSS 13.0 (SPSS, Inc., Chicago, IL). For all statistical tests, a two-tailed *P* value < 0.05 was considered statistically significant.
